# Efficacy and Safety of Intrathecal Magnesium Sulphate as an Adjuvant to Hyperbaric Bupivacaine Versus Isobaric Levobupivacaine in Caesarean Deliveries: A Prospective, Randomized, Double-Blind, Controlled Trial

**DOI:** 10.7759/cureus.90440

**Published:** 2025-08-18

**Authors:** Shravan Kumar Jat, Savita Meena, Sunita Meena, Sandeep Chhipa

**Affiliations:** 1 Anaesthesiology, Sawai Man Singh Medical College and Hospital, Jaipur, IND

**Keywords:** bupivacaine, caesarean section, levobupivacaine, magnesium sulphate, spinal anesthesia

## Abstract

Background

Hyperbaric bupivacaine (HB), commonly used in caesarean sections (CS), may cause cardiotoxicity and high sympathetic blocks. Isobaric levobupivacaine (IL) provides effective sensory block with fewer hemodynamic changes and less toxicity. Adding magnesium sulphate (MS) enhances spinal anaesthesia. This study compared the efficacy and safety of intrathecal 0.5% IL-MS combination with 0.5% HB-MS combination in patients undergoing CS.

Methods

This prospective, randomized, double-blind, parallel-group study involved 74 parturients undergoing CS. The patients were randomly divided into two groups: IL-MS group (n=37, 0.5% IL and 75mg MS) and HB-MS group (n=37, 0.5% HB and 75mg MS). The time to the first request for analgesia was the primary outcome measure. The secondary outcome measures were time to achieve sensory and motor block, time to sensory and motor recovery, and adverse events.

Results

The IL-MS group had a significantly longer time to first rescue analgesia (487 min) compared to the HB-MS group (409 min, p < 0.05). Early sensory block (4 min vs. 5 min, p < 0.01) and motor block onset (5 min vs. 6 min, p < 0.01) were achieved in the IL-MS group compared to HB-MS. Earlier sensory (278 min vs 306 min, p < 0.01) and motor recovery (243 min vs. 268 min, p < 0.01) in the IL-MS group than the HB-MS group were achieved. Adverse events were fewer in the IL-MS group, including no bradycardia cases compared to the HB-MS group.

Conclusion

Intrathecal IL-MS is a useful alternative to HB-MS for patients undergoing CS with longer duration of analgesia and better hemodynamic stability.

## Introduction

The increasing prevalence of caesarean sections (CS) highlights significant challenges in maternal healthcare, with rates increasing in India from 17.2% in 2015-16 to 21.5% in 2019-21 [1,2]. For CS, neuraxial anesthesia, particularly spinal anesthesia, is preferred over general anesthesia due to its enhanced safety and is utilized in up to 96% of cases at tertiary centers [1].

Over the years, various adjuvants, both opioid and non-opioid, have been explored to improve neuraxial analgesia, with ongoing research aimed at identifying alternatives that minimize adverse effects. Recent studies have underscored the efficacy of magnesium sulphate (MS) as an intrathecal adjuvant, demonstrating its ability to prolong analgesia during labor and enhance postoperative recovery by reducing the need for rescue analgesics [3]. Notably, MS provides effective analgesia with a favorable safety profile, without risk of respiratory depression associated with opioid adjuvants [4,5].

Hyperbaric bupivacaine (HB) is a widely employed local anesthetic for CS, yet it carries risks such as sudden cardiac arrest due to sympathetic block extension [1,6]. Conversely, isobaric solutions provide a more predictable distribution of anesthesia. Levobupivacaine, the S(-) enantiomer of racemic bupivacaine, has emerged as a safer alternative, characterized by lower cardiovascular and central nervous system toxicity, as well as a more selective neuraxial blockade with a shorter duration of motor blockade [7].

Prolonged postoperative analgesia supports early mobilization, but extended motor blockade from long-acting local anesthetics may delay ambulation. Studies show earlier motor recovery with isobaric levobupivacaine (IL) compared to HB [8-10]. Addition of intrathecal MS to HB is reported to prolong analgesia [11]. We hypothesized that intrathecal levobupivacaine with MS may extend analgesia duration and promote faster motor recovery than HB, thus encouraging early ambulation. However, in patients undergoing CS, MS as an adjuvant to IL versus HB is rarely evaluated [12,13]. The objective of the study was primarily to compare MS as an adjuvant to either HB or IL regarding duration of analgesia in patients undergoing CS, and secondarily, to determine the sensory and motor block characteristics, hemodynamic characteristics, and adverse effects in mother and child.

## Materials and methods

Study design and ethics

This prospective, randomized, double-blind, parallel-group study was conducted over a period of four months (February 2024 to May 2024) in the Department of Obstetrics and Gynecology of a tertiary care hospital. The study was approved by the Institutional Ethics Committee, SMS Medical College and Attached Hospitals, Jaipur (265/mc/EC/2023, Dated 12 January 2024) and conformed to the Consolidated Standards of Reporting Trials (CONSORT) guidelines. The study was registered in the Clinical Trial Registry of India (CTRI/2024/02/062220). Prior to enrolment, written informed consent was obtained from all the patients.

Population

The study included parturients aged 19-40 years, with a gestational age of 37-42 weeks, American Society of Anesthesiologists (ASA) physical status II, and undergoing CS. Parturients with high-risk pregnancy, any endocrine disorders, twin pregnancy, and those with contraindication for neuraxial nerve block were excluded.

Randomization and blinding

**Figure 1:**
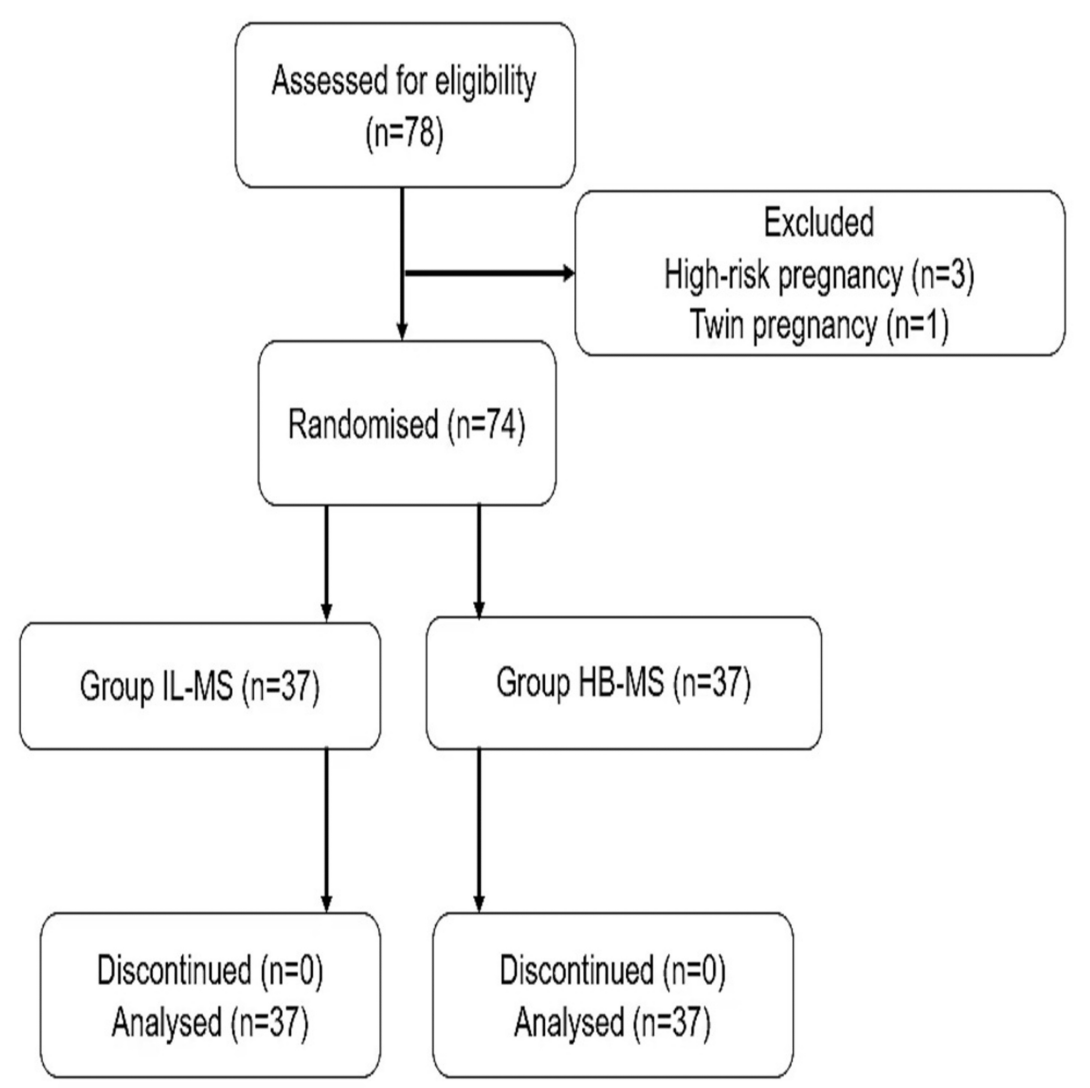
Consolidated Standards of Reporting Trials (CONSORT) flow diagram for the study IL: isobaric levobupivacaine, HB: hyperbaric bupivacaine, MS: magnesium sulphate

A computer-generated randomization table was used, and the patients were randomly and equally allocated in the ratio of 1:1 to one of the study groups (Figure 1). Sequentially numbered opaque envelopes were used to perform allocation concealment. To maintain blinding, neither the anesthetist administering the subarachnoid block nor the patient was aware of the assigned groups or the drugs used. The anesthesiologist who administered the anesthesia was distinct from the one who prepared and provided the drugs.

Study groups

On the day of the surgery, the opaque envelopes were opened, and the patients were randomized to either Group IL-MS (n=37, received 2cc of 0.5% IL combined with 0.15 ml of 50% MS [75-mg]) or Group HB-MS (n=37, received 2 cc of 0.5% HB combined with 0.15 ml of 50% MS [75-mg]).

Sample size calculation

To estimate the expected difference of 1.3 ± 2 minutes in the onset of sensory analgesia between the groups, we considered 80% power, 5% level of significance, and a 95% confidence interval. That resulted in a sample size of 37 parturients per group.

Study protocol

Upon arrival in the operating room, baseline heart rate (HR), blood pressure (BP), and oxygen saturation were recorded. Preloading was done with Ringer lactate at 10 mL/kg per institutional protocol. Spinal anesthesia was administered using a 25G Quincke needle at the L2-L3 or L3-L4 interspace in the sitting position. To ensure blinding, the medication was prepared and injected by an anesthesiologist not involved in the study. HR, non-invasive BP, oxygen saturation, and respiratory rate were monitored, while sensory and motor block levels were assessed every two minutes for the first 10 minutes, then every five minutes for 30 minutes, then every 10 minutes until surgery completion.

The time to achieve loss of sensation to pinprick at the T4 level and motor blockade was determined using the modified Bromage score [14], with the onset of motor blockade marked by Bromage 3. Hemodynamic parameters, including systolic BP, diastolic BP, and mean arterial pressures (MAP), were also monitored. Bradycardia (HR <60 bpm) was managed with IV atropine 0.6 mg, while hypotension (MAP reduction >20% from baseline or MAP <60 mmHg) was treated with IV ephedrine 6 mg and fluids. Nausea and vomiting were treated with IV ondansetron 4 mg.

Study outcomes

The primary outcome measure was duration of analgesia, determined as the time to the first request for analgesia when the visual analogue scale (VAS) exceeded 4 or when the patient requested it. The VAS score ranged from 0 to 10, with 0 indicating no pain and 10 indicating unbearable pain [14]. Patients received IV paracetamol (15 mg/kg) for VAS >4 or upon request. If pain persisted after paracetamol, IV tramadol (100 mg) was administered as rescue analgesia.

The secondary outcome measures were (i) postoperative sensory and motor block characteristics, and their assessment continued until complete recovery of both functions. Sensory recovery time was defined as the time for the sensory block to regress by using two segment regression method, while motor recovery time was marked by achieving Bromage 0; (ii) hemodynamic characteristics, including HR and non-invasive BP; and (iii) adverse effects, including shivering, hypotension, and bradycardia, monitored and recorded at 0, one, three, six, 12, and 24 hours.

Statistical analysis

The categorical and continuous variables are represented as frequency (percentage) and mean ± standard deviation, respectively. A chi-square test and independent sample t-test were used to compare study parameters on various categorical and continuous variables, respectively. The p < 0.05 was considered significant.

## Results

Both HB-MS (bupivacaine) and IL-MS (levobupivacaine) groups were comparable regarding age, body weight, systolic BP, diastolic BP, duration of surgery, as well as APGAR score at one and five minutes (all p > 0.05), while the HR was significantly raised in the IL-MS group (p < 0.001) (Table 1).

**Table 1:** Comparison of demographics and baseline characteristics Data represented as mean ± standard deviation, SBP: systolic blood pressure, DBP: diastolic blood pressure, HR: heart rate, APGAR: appearance, pulse, grimace, activity and respiration, IL: isobaric levobupivacaine, HB: hyperbaric bupivacaine, MS: magnesium sulphate

Variables	Group HB-MS (n=37)	Group IL-MS (n=37)	p
Age, years	26.19 ± 3.31	26.81 ± 3.59	0.442
Weight, kg	58.41 ± 5.86	58.27 ± 5.05	0.913
SBP, mmHg	122.8 ± 10.22	124.5 ± 8.92	0.448
DBP, mmHg	76.95 ± 8.92	80.19 ± 8.1	0.106
HR, minute	97.43 ± 11.76	105.2 ± 5.62	0.000
Duration of surgery, minute	58.95 ± 9.74	58.78 ± 10.3	0.942
APGAR score at 1 minute	8.08 ± 0.68	8.32 ± 0.63	0.975
APGAR score at 5 minutes	9.43 ± 0.50	9.54 ± 0.51	0.982

Time at first request of rescue analgesia was significantly longer in the IL-MS group than the HB-MS group (487 min vs 409 min, p < 0.05) (Table 2).

**Table 2:** Comparison of post-operative analgesia Data represented as mean ± standard deviation, IL: isobaric levobupivacaine, HB: hyperbaric bupivacaine, MS: magnesium sulphate

	Group HB-MS (n=37)	Group IL-MS (n=37)	p
Time at first request of rescue analgesia, minute	409.1 ± 73.29	487.4 ± 152.5	0.006

Time to achieve the highest level of sensory block was significantly earlier in the IL-MS group than the HB-MS group (4 min vs 5 min, p < 0.01). Similarly, the time to achieve complete motor block was significantly earlier in the IL-MS group than the HB-MS group (5 vs 6 min, p < 0.01) (Table 3).

**Table 3:** Comparison of sensory and motor block characteristics Data represented as mean ± standard deviation, IL: isobaric levobupivacaine, HB: hyperbaric bupivacaine, MS: magnesium sulphate

Characteristics	Group HB-MS (n=37)	Group IL-MS (n=37)	p
Time to achieve the highest level of sensory block, minutes	5.36 ± 0.95	4.23 ± 0.72	0.000
Time to achieve complete motor block, minutes	6.22 ± 1.11	4.78 ± 0.89	0.000
Time of two segment regression from highest sensory level, minutes	305.7 ± 32.86	278.2 ± 29.96	0.000
Time to achieve motor recovery, minutes	268 ± 26.14	243.3 ± 26.72	0.000

The time to sensory recovery, time of two segment regression from highest sensory level, was significantly earlier in the IL-MS group than the HB-MS group (278 min vs 306 min, p < 0.01). Similarly, the time to achieve complete motor recovery was significantly earlier in the IL-MS group than the HB-MS group (243 vs 268 min, p < 0.01) (Table 3).

In the HB-MS group, bradycardia, hypotension, and shivering were observed in two, 20, and nine patients, respectively, while in the IL-MS group, corresponding adverse events were observed in 0, 12, and four patients, respectively. The groups had comparable adverse effects profiles (p > 0.05) (Table 4).

**Table 4:** Comparison of adverse events IL: isobaric levobupivacaine, HB: hyperbaric bupivacaine, MS: magnesium sulphate

Adverse effects	Group HB-MS (n=37)	Group IL-MS (n=37)	p
Bradycardia	2 (5.41%)	0 (0%)	NA
Hypotension	20 (54.05%)	12 (32.43%)	> 0.05
Shivering	9 (24.32%)	4 (10.81%)	> 0.05

## Discussion

The principal findings of the study revealed that the IL-MS group had superior efficacy and safety compared to the HB-MS group. The IL-MS group had a longer time to first request of rescue analgesia, faster onset of sensory and motor blocks, and earlier recovery of sensory and motor functions. The IL-MS group demonstrated no bradycardia, lower incidence of hypotension and shivering compared to the HB-MS group although not statistically significant.

In this study, the time to first request of rescue analgesia was significantly longer in the IL-MS group compared to the HB-MS group, indicating extended pain relief with IL. This difference can be attributed to the pharmacokinetic and pharmacodynamic profiles of the anesthetics. IL has a slower onset and a more gradual decline in analgesic effect, leading to prolonged pain relief. Conversely, HB typically provides a faster onset but shorter analgesic duration. These findings align with earlier reports suggesting that HB offers quicker onset and prolonged analgesia, while IL demonstrates a superior safety profile [8,10,12]. However, Deepa et al. observed comparable postoperative analgesia durations with both agents [12]. In contrast, Upadhya et al. noted a longer analgesia duration with isobaric bupivacaine compared to HB, although the difference lacked statistical significance [15].

The early onset of sensory blockade in the IL-MS group compared to the HB-MS group may be attributed to the properties of IL. Its density, similar to cerebrospinal fluid, likely promotes uniform intrathecal distribution, enabling faster sensory onset. Supporting these findings, Guler et al. reported a quicker sensory block onset with IL compared to HB in a similar population [8]. However, Duggal et al. observed no significant difference in sensory blockade onset between plain levobupivacaine and HB [16]. Studies suggest that adding MS to HB may delay sensory blockade onset [17]. Buvanendran et al. evaluated intrathecal MS (50 mg) combined with fentanyl (25 mcg) versus plain fentanyl for labor analgesia. They also assessed the baricity of MS-fentanyl mixtures, finding them slightly hypobaric relative to cerebrospinal fluid, which could influence the sensory block characteristics [5].

Similarly, the earlier onset of motor blockade in the IL-MS group compared to the HB-MS group contrasts with Deepa et al., who reported comparable results between the two anesthetics [12]. In contrast, Guler et al. found delayed onset of motor blockade in the group receiving levobupivacaine with fentanyl compared to bupivacaine with fentanyl for spinal anesthesia in parturients undergoing CS [8]. This difference could be attributed to the higher density of hyperbaric bupivacaine, which facilitates rapid spread within the cerebrospinal fluid, and quicker nerve root blockade responsible for motor function. Isobaric levobupivacaine, while effective, exhibits a slower onset due to its density similar to cerebrospinal fluid, leading to gradual distribution and less pronounced motor blockade. However, in our study, the synergistic effects of MS with IL appeared to enhance the analgesic properties of local anesthetics and may have accelerated the onset of both sensory and motor blocks.

In this study, sensory recovery time was shorter in the IL-MS group compared to the HB-MS group, suggesting that IL facilitates faster sensory recovery in CS patients undergoing spinal anesthesia. Sensory recovery was assessed by the return of pinprick sensation at the T12 level, while analgesia duration was evaluated using the VAS scale or patient request for analgesia, which is a subjective measure [18]. This explains the observed differences between sensory recovery and postoperative analgesia duration. Our findings align with those of Guler et al. [8] and Deepa et al. [12], who reported early sensory and motor recovery in patients receiving IL compared to HB in CS.

In this study, parturients receiving IL-MS experienced significantly shorter motor blockade recovery compared to those receiving HB with MS, indicating faster motor function return in the IL-MS group. This could be attributed to the lower systemic absorption and localized action of IL, leading to a shorter motor blockade duration. These findings align with various studies [8-10,12,19]. Moreover, studies have also demonstrated that levobupivacaine and ropivacaine result in earlier motor blockade regression compared to bupivacaine [20].

The occurrence of adverse events, including bradycardia, hypotension, and shivering, highlight distinct hemodynamic profiles of the two anesthetic agents. The absence of bradycardia in the IL-MS group suggests that IL with MS provides a more stable hemodynamic profile. HB is known to cause pronounced sympathetic blockade, leading to bradycardia and hypotension, a phenomenon supported by our finding of higher hypotension incidence in the HB-MS group. Sympathetic blockade caused by hyperbaric solutions leads to vasodilation and reduced venous return, contributing to hypotension [21]. This aligns with studies by Deepa et al., who reported no bradycardia and comparable hypotension rates between groups [12], and Guler et al., who observed significantly lower hypotension (16.6%) with levobupivacaine compared to bupivacaine (36.6%), attributing this to reduced local anesthetic doses and added fentanyl [8].

The low incidence of shivering in the IL-MS group is noteworthy. Shivering during spinal anesthesia can result from hypothermia, loss of sympathetic tone, or anesthetic effects [22]. The low incidence of shivering in the IL-MS group may suggest that the MS used in conjunction with levobupivacaine may allay thermoregulation response. Our findings contrast with Deepa et al., who reported similar incidence of shivering between the groups [12], while Rao et al. reported no shivering in the levobupivacaine group compared to bupivacaine [19].

Despite the randomized-controlled and double-blinded design, the study had a few limitations. Firstly, neonatal outcomes were not assessed using cord blood pH, limiting the evaluation of anesthetic techniques on fetal well-being. Secondly, differences in baricity between the local anesthetics could have influenced the results, as hyperbaric and isobaric solutions vary in block spread and duration. Thirdly, the sample size may have been insufficient to detect subtle outcome differences, indicating the need for larger cohort studies. Fourth, the study did not investigate long-term maternal or neonatal effects, which could provide valuable insights into safety and efficacy. Lastly, variations in administration techniques or patient characteristics may have introduced confounding factors, potentially affecting generalizability of the findings to broader populations.

## Conclusions

The IL-MS provides significant advantages over HB-MS in terms of analgesic efficacy and safety during spinal anesthesia for CS. The IL-MS group demonstrated a longer duration before the first request for rescue analgesia, quicker onset of sensory and motor blocks, and faster recovery of sensory and motor functions. Additionally, the adverse effects profile of the IL-MS group differed from that of HB-MS group with no bradycardia, low incidence of hypotension and shivering.
